# Investigation into Hypoglycemic, Antihyperlipidemic, and Renoprotective Potentials of* Dennettia tripetala* (Pepper Fruit) Seed in a Rat Model of Diabetes

**DOI:** 10.1155/2017/6923629

**Published:** 2017-10-17

**Authors:** Innocent Anioke, Chukwugozie Okwuosa, Ikenna Uchendu, Olive Chijioke, Ogechukwu Dozie-Nwakile, Ifeoma Ikegwuonu, Peculiar Kalu, Maryann Okafor

**Affiliations:** ^1^Department of Medical Laboratory Sciences, Faculty of Health Sciences and Technology, College of Medicine, University of Nigeria, Enugu Campus, Enugu, Nigeria; ^2^Department of Chemical Pathology, College of Medicine, Nnamdi Azikiwe University, Nnewi, Nigeria

## Abstract

This study investigated the hypoglycemic, antihyperlipidemic, and renoprotective potentials of* Dennettia tripetala* (DT) in a rat model of diabetes. The hypoglycemic activity in crude methanol seed extract of DT (CMEDT) and methanol seed fraction of DT (MFDT) measured by glucose oxidase method was increased by 47.37% and 28.72%, respectively, after 8 hours of administration. After 10 days of treatment, CMEDT and MFDT gave a good glycemic control with the highest percentage reduction of 75.82% and 71.34% in glucose level, respectively, which is closely compared with 79.91% in glibenclamide. Using the enzymatic assay and Friedewald's equation, there was a significant reduction in serum level of total cholesterol (TC), triglyceride (TG), very-low-density lipoprotein (VLDL), and low-density lipoprotein (LDL) and a significant increase in high-density lipoprotein (HDL) (*p* < 0.05) following treatment with CMEDT and MFDT, when compared with the untreated group, although results varied in dosed groups, with high dose of MFDT showing a better lipid-lowering activity. High dose of MFDT improved lipid metabolism and increased percentage protection against atherogenesis by 44%. However, neither CMEDT nor MFDT ameliorated the renal biochemical alteration in urea and creatinine. Thus, the study demonstrates hypoglycemic and antihyperlipidemic potentials of DT seed in diabetes.

## 1. Introduction

Diabetes mellitus (DM) is a chronic metabolic disorder of impaired carbohydrates, fat, and protein metabolism, characterized by hyperglycemia, polyuria, polydipsia, weight loss, polyphagia, and glycosuria due to insulin deficiency. This could be secondary to reduced insulin production by the pancreas or lack of response to insulin by target tissues such as the liver, adipose tissue, and skeletal muscle [1, 2]. Typical metabolic derangement in diabetes involves decreased utilization of carbohydrates, excessive glycogenolysis, and increased gluconeogenesis from amino acids and fatty acids [3, 4]. Diabetes may be acquired or hereditary.

In 2010, about 285 million diabetic cases were reported, representing 6.4% of the adults globally, and this is expected to increase to about 439 million adults (7.7%) by 2030 [5]. In the past decades, the prevalence of DM in Nigeria was 2.2% [6]. In the recent past, this has increased to 5.0% [7] whereas about 6.7% prevalence rate of type 2 diabetes has been documented in Southeast Nigeria [8]. In Africa, where access to quality healthcare services is limited, Nigeria specifically has the highest burden of DM [7, 9, 10]. For instance, Ogbera et al. [9] reported that DM cases account for approximately 15% of all medical admissions and 22% of all medical deaths in Nigerian hospitals. This evidence demonstrates a critical health challenge for DM cases in Nigeria.

Along with hyperglycemia (which causes glycation of proteins) and dyslipidemia, diabetes is associated with micro- and macrovascular complications affecting several organs of the body [11–13]. All DM patients have at least one type of dyslipidemia whereas others have combined dyslipidemia, which is characterized by a reduction in high-density lipoprotein (HDL) level and elevation in low-density lipoprotein (LDL) and triglyceride (TG) levels [10, 14]. These lipoprotein disturbances constitute a major risk factor for coronary heart disease (CHD) which is a cause of morbidity and mortality in patients [15]. While there are various pathophysiological mechanisms leading to diabetic nephropathy (DNP) [16–19], the formation of advanced glycation end products induced by chronic hyperglycemia and dyslipidemia is considered to play a vital role in the progression of DNP [20, 21].

Given that DM is a multifactorial disease, the treatment strategy should focus not only on maintaining a tight control of blood glucose level within the normal limit, but also on correcting the associated metabolic defects [22] such as dyslipidemia, all of which are implicated in the development of DNP [23–25].

During the past decade, herbal medicine has been gaining popularity exponentially both in developing and in developed countries. As such, in traditional practice, in many countries, herbal remedies have been employed in treatment and management of DM [26]. Perhaps, as herbal drugs have been associated with minimal side effects besides their perceived effectiveness [27, 28], the WHO recommends their use for the treatment of DM [29]. Furthermore, according to the Indian Council of Medical Research (ICMR), there is no satisfactory treatment yet for DM using allopathic drugs, which therefore underpins the investigation of suitable herbal therapy [26, 30].

**Figure 1:**
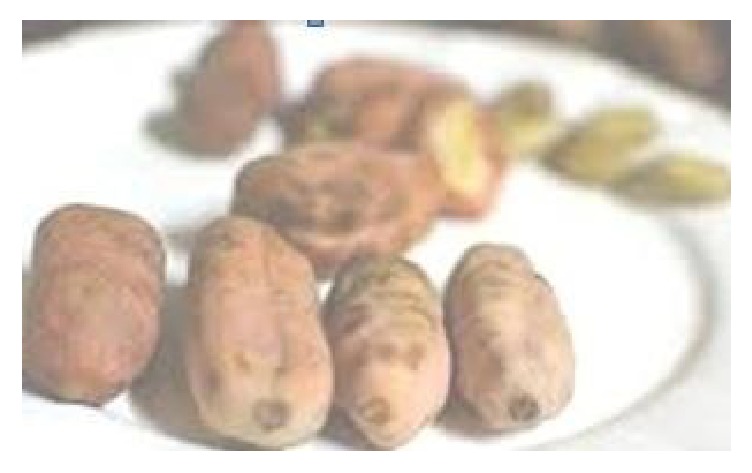
*Dennettia tripetala* fruits.

Unsurprisingly, there are many herbal remedies suggested for the treatment of diabetes [31–33], which clinical investigations are yet to confirm most, for their efficiency and safety. One such dietary plant product is* Dennettia tripetala* (Annonaceae) (Figure 1), which is widely consumed in Southern Nigeria. It is found in the tropical rainforest region of Nigeria and sometimes in Savana areas [34]. Locally, it is known as “Nkarika” (in Efiks of Calabar), “Nmimi” (in Igbo), and “Igbere” (in Yoruba). The mature fruits have a spicy taste and constitute the main edible portions [35]. The leaves and roots are utilized, in addition to the fruits, for medicinal purposes, especially in the southern part of Nigeria [36].

*Dennettia tripetala* (pepper fruit) is believed to be endowed with hypoglycemic potentials. However, there is no documented report to the best of the researchers' knowledge on the antidiabetic and specifically on the antihyperlipidemic effect of the seed extract of* D. tripetala* at the time of this study. Therefore, the study was designed to investigate the hypoglycemic, antihyperlipidemic, and renoprotective effect of* Dennettia tripetala* (DT) seed using a rat model of diabetes. The outcome of this study would eventually be useful in the management of DM in humans later on, if found to reverse major metabolic alterations (e.g., hyperglycemia, hyperlipidemia, and abnormal renal biochemistry) associated with DM.

## 2. Materials and Methods

### 2.1. Collection and Authentication of Plant Material

Fresh samples of ripe and unripe* D. tripetala* fruits were purchased from the main market in Enugu, Enugu State, Nigeria. The plant material was authenticated by a consultant taxonomist at the herbarium section of the Department of Plant Science and Biotechnology, University of Nigeria, Nsukka, and a voucher specimen was deposited at the herbarium with reference number UNH 8^C^ for future reference.

### 2.2. Processing of* Dennettia tripetala* Seed Powder

*Dennettia tripetala* seeds were steeped in water for 24 hours to loosen the bark. After dehulling, the seeds were washed three times with clean water to remove debris and sand and then dried under the shade (below 40°C). The dried seeds were milled with an electric blender and finally ground into powder using a hammer mill (500# grinder/Fuyu Metal, Linyi Fuyu Metal Products Co., Ltd., China) and thereafter passed through a 200 × 50 mm sieve (Retsch GmbH, Germany).

### 2.3. Chemicals and Reagents

Streptozotocin was purchased from Sigma-Aldrich Ltd., United Kingdom (UK), while Clamide (glibenclamide) manufactured by Hovid Berhad (121 Jalan Tunku Abdul Rahman, 30010 Ipoh, Malaysia) was purchased from Enugu. The commercial kits for creatinine, urea, and lipid profiles estimation were purchased from Randox Laboratory, United Kingdom. Other reagents and chemicals were obtained from research laboratories in Enugu. All the chemicals used were of analytical grade, while the water was glass-distilled.

### 2.4. Preparation of the Crude Methanol Extract and Fraction

The powdered seeds (1500 g) of* Dennettia tripetala* were weighed and macerated in 2.5 liters of absolute methanol in a 10-liter container and left for 48 hours. The mixture, intermittently, was agitated during the extraction process. After 48 hours, the mixture was sieved using muslin cloth and filtered with Whatman No. 1 filter paper and the filtrate then evaporated to dryness on a rotary evaporator. The residue obtained was labeled as the crude methanol extract (CMEDT) and stored in a refrigerator at 4 ± 2°C until required. The marc was allowed to air-dry on McIntosh. The dried marc was reweighed and further extracted in N-hexane and ethyl acetate solvents, respectively, in that order of increasing solubility gradient. The marc obtained after the extractions was spread out on McIntosh and allowed to air-dry in a rotary evaporator (BUCHI Rotavapor R-215, Switzerland). The dried marc was reweighed and macerated in 2.5 liters of methanol for 48 hours. After 48 hours, the mixture was treated in a similar fashion as above while the residue obtained was labeled as methanol fraction (MFDT) and kept in a refrigerator at 4 ± 2°C until required.

### 2.5. Determination of the Extractive Value for the Crude Methanol Seed Extract and Fractions

The concentrations of the CMEDT and MFDT were determined by evaporating 1 ml each of the residue in an evaporating dish of known weight in an oven (Gallen Kamp, UK) to dryness at 70°C. The dish containing the residue was allowed to cool and the weight of the residue was obtained by subtracting the weight of the empty dish from the weight of the dish and residue. The above process was done in duplicate, and the average weights were taken. The dry residue was then weighed to obtain the concentration which is expressed in g/ml. The residues of CMEDT and MFDT gave a concentration of 0.6 g/ml and 0.8 g/ml, respectively. The appropriate concentration then was calculated and reconstituted with water prior to administration.

### 2.6. Phytochemical Analysis

Standard procedures as described by Sofowora [37], Trease and Evans [38], and Harborne [39] were used to identify the bioactive chemical constituents present in DT seeds using appropriate chemical tests for the screening and identification process.

### 2.7. Experimental Animals

#### 2.7.1. Animal Housing and Management

A total of 56 apparently healthy albino rats of same sex and age weighing between 100 g and 150 g were obtained from Animal House of the College of Medicine, University of Nigeria Teaching Hospital, Enugu. The rats were divided into seven (7) groups of eight (8) rats each. They were acclimatized for a period of two (2) weeks in clean gauzed cages according to their body weight (*x* ± 20 g) under good laboratory conditions at the Animal House of the College of Medicine, University of Nigeria, Enugu Campus. They were also fed with commercial standard pellets (Topfeed®, Nigeria) and clean water was provided daily ad libitum. Handling, management, and use of animals for the experiment followed the “Ethical and Scientific Considerations Regarding Animal Testing and Research” [40].

### 2.8. Acute Toxicity (Median Lethal Dose, Ld_50_)

The oral median lethal dose of CMEDT and MFDT was determined in rats as described by Lorke [41].

### 2.9. Induction of Diabetes

Streptozotocin (100 mg) was freshly dissolved in 1 ml of 0.1 M citrate buffer (pH 4.5) and was administered to various groups, based on the body weight of the rats and at a dosage of 45 mg/kg body weight. The administration was done subcutaneously in vehicle volume of 0.07 ml using a syringe with fine needle [42]. A rest period of two days (48 hours) following induction of diabetes was allowed for the blood glucose level to stabilize. During this period, the animals had free access to both water and food. Blood glucose was measured by a glucometer (One Touch Ultra®, LifeScan, USA), and only rats with blood glucose > 200 mg/dl were considered hyperglycemic. The hyperglycemic rats (*n* = 30) were divided into 6 groups comprising 5 rats in each group for the study.

### 2.10. Clamide (Glibenclamide)

Clamide acts on the *β*-cell membrane leading to the enhancement of the calcium flux across it, hence provoking the brisk release of insulin from the pancreas. Although the insulinemic action of Clamide declines after chronic administration, however, improvement in glucose tolerance is maintained, suggesting its efficient hypoglycemic action [43]. Clamide (glibenclamide) (5 mg × 20), dissolved in distilled water and made up to 50 ml giving a concentration of 2 mg/ml, was used in the study as a standard antidiabetic drug.

### 2.11. Experimental Design

The rats were divided into the following groups:

  Group A received 50 mg/kg body weight of CMEDT.
  Group B received 100 mg/kg body weight of CMEDT.
  Group C received 50 mg/kg body weight of MFDT.
  Group D received 100 mg/kg body weight of MFDT.
  Group E (diabetic positive control) received 10 mg/kg glibenclamide, the standard DM drug.
  Group F (diabetic negative control) received the vehicle for reconstitution.
  Group G was normal control.

### 2.12. Acute Study

All the diabetic animals were made to fast overnight (12 hrs) and fasting blood glucose level was determined (at 0 hrs) before which the extracts and fraction were administered through the oral gavages. The blood glucose concentration was estimated at 2 hrs, 4 hrs, and 8 hrs, respectively, after the administration for the acute study.

### 2.13. Subacute Study

The second phase (subacute study) began with oral gavages of the extract and fraction twice daily for ten (10) days. Body weights were measured on the 0th day and 10th day of the experiment. Fasting blood glucose was estimated on the 5th day and 10th day, respectively. After the 10th day of treatment with DT, the experiment was terminated and blood samples were collected.

### 2.14. Collection of Blood from Animals

The blood sample used for biochemical analysis was collected through the median canthus of the eye under ether anesthesia using capillary tube into plain tubes. The blood was centrifuged to separate serum for estimation of glucose, lipid profile, urea, and creatinine.

### 2.15. Biochemical Analyses

Blood glucose level was measured using the glucose oxidase method [44].

Renal biochemical parameters: creatinine was estimated by Jaffe method while urea was measured by diacetyl monoxime (DAM) method.

Measurement of serum lipid profile was carried out using enzymatic end point (kit) methods:

  Triglycerides: glycerol-phosphate oxidase (GPOD) method [45]
  Total cholesterol: cholesterol oxidase method (CHOD) [46] and HDL cholesterol: precipitation methods [47]

Friedewald equation was applied for low-density lipoprotein (LDL) cholesterol and very-low-density lipoprotein (VLDL) estimation: LDL cholesterol = total cholesterol − (triglycerides/5 + HDL cholesterol), where TG/5 = VLDL.

Atherogenic index and percentage protection of DT were estimated using the formulae described by Ng et al. [48]:

(1) Atherogenic  index AI=total  cholesterol−HDL  cholesterolHDL;Protection %=AI control−AI treatedAI control×100.

### 2.16. Data Analysis

Data were collected using an appropriate laboratory test method for each biochemical parameter as described above. All data were analyzed using SPSS software (version 22) and results were expressed as mean ± SEM. One-way analysis of variance (ANOVA) followed by post hoc multiple comparison tests was used to compare group means. *p* < 0.05 was considered to be a statistically significant value.

## 3. Results

### 3.1. Acute Toxicity Test

There was no mortality or any signs of behavioral changes or toxicity observed after the administration, suggesting that CMEDT and MFDT have a high degree of safety in Wistar albino rats.

### 3.2. Blood Glucose and Body Weight

Table 1 shows that, in the treatment groups A and C, there was a significantly decreased (*p* < 0.05) plasma glucose level after 8 hours of extract administration when compared with 0 hrs. Although the percentage reduction was the highest in group A (47.37%) as opposed to group E (33.70%), however, it still indicates a potential antihyperglycemic action of CMEDT which may be similar to the standard antidiabetic drug, glibenclamide.

In Table 2, after day 10, there were significantly decreased (*p* < 0.05) plasma glucose levels in A, B, D, and E when compared with F (untreated control), respectively. However, there were no significant differences (*p* > 0.05) in plasma glucose levels when dosed groups were compared with the normal control (G), respectively. This indicates a good glycemic control of CMEDT and MFDT, given that reduction in plasma glucose is closely the same as that of nondiabetic group G. Group A showed the highest percentage reduction (75.82%) in blood glucose level followed by group D (71.34%) which is pretty the same as group E (79.91%) treated with glibenclamide. This indicates an efficient hypoglycemic action of CMEDT and MFDT. In relation to final body weights, there was a significant increase in body weights in groups A and D when compared with the negative (untreated) control, group F.

### 3.3. Serum Lipid Profile

Taken together, in Table 3, a significant reduction in serum level of TC, TG, VLDL, and LDL and a significant increase in HDL were observed following treatment with CMEDT and MFDT, though results varied in dosed groups. High-dose MFDT (group D) showed similar results to glibenclamide, a standard antidiabetic drug (group E), in all parameters except in TC which did not significantly decrease as opposed to the untreated group F. With low-dose CMEDT (group A), other parameters except HDL and LDL showed a similar trend to glibenclamide (group E). This indicates that low-dose CMEDT and more specifically high-dose MFDT have potential antihyperlipidemic activity which is closely compared with that of glibenclamide, the standard antidiabetic drug.

### 3.4. Atherogenic Index and Percentage Protection

Table 4 shows atherogenic index (% protection) of 2.33 (39%) and 2.15 (44%) at doses of 100 mg/kg body weight CMEDT (group B) and 100 mg/kg body weight MFDT (group D), respectively. In group D, percentage protection (44%) was found to be closely compared with that of glibenclamide (group E) at 10 mg/kg body weight (43%), suggesting that DT may possess an antiatherogenic potential that is similar to glibenclamide.

### 3.5. Urea and Creatinine

In Table 5, the mean urea levels in groups D and E were significantly increased (*p* < 0.05) when compared with untreated group F, respectively, while creatinine in all dosed groups showed no significant difference (*p* > 0.05) when compared with group F. This suggests that treatment with DT may not positively protect against DNP.

### 3.6. Phytochemistry Analysis

In Table 6, the phytochemical analysis revealed the presence of bioactive phyto- and biochemical components that adapt DT for its nutritional and therapeutic purposes.

## 4. Discussion

In DM, the major metabolic derangements resulting from insulin deficiency or insensitivity are impaired glucose utilization and altered lipid and protein metabolism [15, 49]. Consequently, the outcome of rapid mobilization of triglycerides, for instance, is usually the elevated levels of free fatty acids in the plasma. This reduces glucose uptake and suppresses glucose metabolism in peripheral tissues (adipose tissue and skeletal muscle), further exacerbating hyperglycemia [49]. The driving force behind this metabolic alteration of these major energy molecules is attributed to the changes in the activities of molecular targets (or specific proteins or enzymes) involved in glucose/lipid metabolism or glucose transport in target tissues [50]. For instance, impaired glucose utilization and insulin deficiency decrease the expression of a number of proteins such as glucokinase and the GLUT4 glucose transporter gene required for normal response to insulin in liver and adipose tissue, respectively [49].

Antidiabetic drugs improve glucose homeostasis through modulation of these molecular targets [51, 52]. Unfortunately, most synthetic agents such as sulfonylureas (e.g., glimepiride) and meglitinides (e.g., repaglinide) impact a single target [53], without producing a balanced therapeutic effect addressing the cascade of interrelated metabolic derangements such as hyperlipidemia [54]. However, thiazolidinediones (e.g., pioglitazone), a relatively new class of drugs, improve insulin sensitivity in target tissues by binding to and altering the function of a nuclear receptor, peroxisome proliferator-activated receptor-gamma (PPAR-*γ*) [52]. PPAR-*γ* regulates the transcription of specific proteins involved in glucose metabolism (e.g., glucokinase and GLUT4 glucose transporter protein) and lipid metabolism (e.g., lipoprotein lipase and fatty acid transporter protein), as well as energy balance [15, 50–52]. This suggests that thiazolidinediones as a single agent have the capacity to impact glucose and lipid metabolism [55]. Most of these popularly prescribed antidiabetic therapies have an excellent safety profile. However, their associated adverse effects, such as, but not limited to, headache, dizziness, diarrhea, nausea, and dyspepsia, vitamin B12 and folic acid deficiency, weight gain, hypoglycemia, fluid retention, and edema [51–53], are thought to be clinically relevant.

Therefore, the need for alternative complimentary agents with holistic therapeutic potential and minimal side effects has been an important growing consideration in DM management over the past decade [56]. Moreover, effective oral antidiabetic drugs should have an impact on lipid metabolism beyond the expected effects on glycemic control [56]. Additionally, Unnikrishnan et al. [54] underpinned the need to identify natural agents with a more holistic mechanism of action, without disturbing the physiological equilibrium of the patients.

In light of the current study, DT has emerged as a potential alternative antidiabetic agent from natural origin with such therapeutic impact. The use of a single natural agent such as DT will possibly ensure increased patient convenience and the potential for increased compliance to therapy. DT may have exerted a multimodal effect, which is moderate and spreads over different targets tissues, thereby modulating several cascading effects on glucose and lipid metabolism triggered by DM, to improve glucose homeostasis.

### 4.1. Effect of DT on Blood Glucose and Body Weight

In the present study, treatment with low-dose CMEDT (50 mg/kg body weight) and high-dose MFDT (100 mg/kg body weight) produced a significant decrease in plasma glucose level closely compared to glibenclamide at 10 mg/kg body weight. Glibenclamide acts on the *β*-cell membrane which enhances calcium influx across it, thereby provoking the brisk release of insulin from the pancreas leading to increased glucose utilization and hence decreased plasma glucose [43, 57, 58]. The phytochemical investigation of DT seed carried out in the current study revealed the presence of saponins, resins, alkaloids, and so forth. Studies in the recent past [59–64] reported that dietary flavonoids, alkaloids, saponins, tannins, and glycosides have antidiabetic potentials. These bioactive phytochemicals, as reported in previous studies [65–68], may either singly or in synergy with one another be responsible for the significant glucose-lowering activity reported in the current study. Although the precise mechanism leading to the hypoglycemic effect was not elucidated in the study, the fact that the percentage glucose reduction noted at low-dose CMEDT (75.82%) and high-dose MFDT (71.34%) was closely compared with glibenclamide (79.91%) suggests that the mechanism of action of DT and glibenclamide may be similar, which may be attributed to increased secretion of insulin from the *β*-cells of the pancreas. Additionally, other mechanisms may have played a role in the glucose-lowering effect following treatment with DT. For instance, glucose kinase (GK) is known to regulate as well as facilitate the glucose uptake/utilization in hyperglycemic state in the liver [14, 69]. Grimsby et al. [70] suggested that, with the increase in GK activity, glucose utilization is often stimulated, leading to an increase in glycogen synthesis which in turn results in a significant plasma glucose reduction. A study in the recent past [71] underpinned the notion that hypoglycemia results with GK activator, as GK activity is increased with simultaneous conversion of plasma glucose to liver glycogen in diabetic patients. Although the activity of hepatic GK is not investigated in the current study, however, we suggest that DT could be a potential liver glucose kinase activator.

In streptozotocin-induced diabetic rats, body weight decreased significantly compared to the normal control, which may be attributed to increased degradation of structural protein due to damage to the intracellular signaling pathways implicated in maintaining the balance between protein synthesis and degradation [72, 73]. Interestingly, there was a significant increase in the body weight when compared with the untreated (negative control) group after treatment. This suggests that DT may have a bioactive potency similar to IGF-1, which increases protein synthesis in diabetes to restore muscle wasting through the activation of Akt/mTOR pathways [74–77].

### 4.2. Effect of DT on Plasma Lipid Profile

According to documented evidence [78], abnormalities in insulin action or deficiency rather than chronic hyperglycemia per se significantly lead to increased plasma TC and TG seen in diabetic patients. In the absence of insulin, hypertriglyceridemia normatively occurs given that lipoprotein lipase (LpL) is not activated, an enzyme which is needed to hydrolyze TG [14, 79]. There is closely linked bidirectional cholesterol exchange between HDL particle and TG-rich lipoproteins [14]. Kameswararao et al. [80] reported that maintenance of tight glycemia control results in significant reversal of lipid abnormalities associated with drug-induced diabetes in rats.

Interestingly, in the current study, the results indicate that low-dose CMEDT and more specifically high-dose MFDT reversed the diabetic dyslipidemia characterized with increased TG and decreased HDL associated with streptozotocin-induced diabetic rats noted in the diabetic control (untreated) group F. High-dose MFDT, however, showed a more effective antidyslipidemic potential closely compared with that of glibenclamide. This may be attributed to the brisk release of insulin from the pancreas activating the LpL which reduces TG through hydrolysis. Furthermore, the results suggest that DT may have reversed increased free fatty acids which affect adequate glucose metabolism in diabetic patients [81, 82]. Presence of insulin stimulates fatty acids biosynthesis, incorporates fatty acids into TG in the liver and adipose tissue [83] by inhibiting hormone sensitive lipase (HSL), increases utilization of glucose, and decreases mobilization of free fatty acids from the fat depositions [84]. Apart from the aforementioned mechanism, it is speculated that DT may have inhibited the activity of plasma endothelial lipase (EL) involved in HDL metabolism, despite being not studied, thereby decreasing the phospholipase action of EL which derives HDL catabolism by hydrolyzing its phospholipids content [85–88]. Furthermore, the increase in HDL fraction may also be attributed to the inhibition of cholesteryl ester transfer protein (CETP) by DT, an enzyme that regulates the transfer of cholesterol ester from HDL to other fractions of plasma cholesterol, particularly TG lipoproteins [89]. It is possible that CMEDT and MFDT like some glucose-reducing agents (e.g., thiazolidinediones) may have improved insulin action on peripheral tissues leading to a greater improvement in lipid profile parameters [90, 91] reported in the current study.

Growing evidence suggests that diabetic dyslipidemia is closely associated with cardiovascular risk [14]. According to Panagiotakos et al. [92], diabetic patients at risk of cardiovascular diseases could be predicted using LDL/HDL ratio. This is referred to as the atherogenic index (AI), with an index of greater than 5 set as the cut-offs for at-risk patients [48]. Therefore, given that values of CMDT and MFDT in all dosed groups were less than 5 (<5), it may be inferred that DT seeds have a cardiovascular protective potential besides its antihyperlipidemic potency.

### 4.3. Effect on Renal Biochemical Parameters

Following the induction of diabetes, the concentration of urea was significantly increased in untreated rats when compared with the normal control rats, suggesting renal involvement. Surprisingly, treatment with either CMDT or MFDT, at high or low dose, in all dosed groups was not found to produce significant reduction in urea when compared with the untreated group (diabetic control). Creatinine concentration showed no significant difference after treatment either, when compared with the untreated diabetic groups. This suggests that treatment with DT may not positively protect against diabetic nephropathy (DNP).

## 5. Conclusion

The present experimental study demonstrated hypoglycemic and antihyperlipidemic potency of* Dennettia tripetala* (pepper fruit) (DT) which is closely compared with glibenclamide in the treatment of diabetes. However, there is no evidence of renoprotective potential associated with DT in the study. The potential antidiabetic properties linked with DT need to be therapeutically maximized to ameliorate the burden of diabetes and its complications in the society. There is, therefore, the need for further investigation to elucidate the phytochemical component(s) present in DT seed with the antidiabetic property observed in the study.

## Figures and Tables

**Table 1:** Hypoglycemic (acute) effect of crude methanol seed extract and fraction (CMEDT and MFDT) in streptozotocin-induced diabetic rats.

Group	Treatment (twice daily)	Plasma glucose concentration (mg/dl)				% reduction
		0 hours	2 hours	4 hours	8 hours	
A	CMEDT 50 mg/kg body weight	413.6 ± 39.44	315.60 ± 9.55	320.80 ± 10.29	217.60 ± 12.00	47.37^*∗*^
B	CMEDT 100 mg/kg body weight	326.60 ± 19.84	377.20 ± 6.86	372.40 ± 34.54	335.60 ± 4.65	−2.76
C	MFDT 50 mg/kg body weight	563.40 ± 28.43	413.40 ± 7.59	432.60 ± 29.15	401.60 ± 62.22	28.72^*∗*^
D	MFDT 100 mg/kg body weight	367.80 ± 21.26	455.20 ± 64.25	522.20 ± 60.81	329.60 ± 29.15	10.39
E	Glibenclamide 10 mg/kg body weight (positive control)	517.80 ± 32.66	451.60 ± 25.39	441.40 ± 16.90	343.40 ± 0.87	33.70^*∗*^
F	Negative (untreated) control	342.00 ± 37.15	446.40 ± 45.46	523.60 ± 32.35	487.80 ± 37.24	−42.63
G	Normal control	104.00 ± 6.79	90.40 ± 1.63	101.60 ± 1.03	87.20 ± 4.57	16.15

Values are mean ± SEM; *n* = 5; ^*∗*^% reduction in plasma glucose level as compared to zero hours after 8 hours.

**Table 2:** Subacute effect of crude methanol seed extract and fraction (CMEDT and MFDT) on plasma glucose level and body weight in streptozotocin-induced diabetic rats.

Group treatment (twice daily)	Initial body weight (g) Day 0	Final body weight (g) Day 10	Plasma glucose concentration (mg/dl)			% reduction	
			Day 0	Day 5	Day 10	Body weight	Glucose level
A: CMEDT 50 mg/kg body weight	142.00 ± 5.83	90.00 ± 4.47^*∗∗*^↑	413.60 ± 39.44	117.20 ± 24.01	100.00 ± 3.67^*∗∗*^↓	36.62	75.82^a^

B: CMEDT 100 mg/kg body weight	124.00 ± 6.78	66.00 ± 8.12	326.60 ± 19.84	175.20 ± 47.28	183.60 ± 38.21^*∗∗*^↓	46.77	43.78^a^

C: MFDT 50 mg/kg body weight	104.00 ± 2.45	60.00 ± 5.48	563.40 ± 28.43	344.00 ± 16.33	327.60 ± 19.42	42.31	41.85^a^

D: MFDT 100 mg/kg body weight	122.00 ± 7.35	94.00 ± 4.00^*∗∗*^↑	367.80 ± 21.26	242.40 ± 56.58	105.40 ± 11.27^*∗∗*^↓	22.95	71.34^a^

E: glibenclamide 10 mg/kg body weight (positive control)	114.00 ± 2.45	86.00 ± 4.00	517.80 ± 32.66	344.00 ± 2.45	104.00 ± 2.07^*∗∗*^↓	24.56	79.91^a^

F: negative (untreated) control	90.00 ± 4.47	66.00 ± 2.45^*∗*^↓	342.00 ± 37.15	342.20 ± 20.32	414.20 ± 26.28^*∗*^↑	26.67	−21.11

G: normal control	124.00 ± 6.78	122.00 ± 3.74	104.00 ± 6.79	75.00 ± 3.27	89.80 ± 5.59	1.61	13.65

*F*-ratio	9.107	19.484	27.475	13.625	44.391		

*p* value	0.000	0.000	0.000	0.000	0.000		

Values are mean ± SEM; *n* = 5; ^*∗*^*p* < 0.05 when compared with normal control; ^*∗∗*^*p* < 0.05 when compared with negative (untreated) control; ^a^% reduction in plasma glucose level as compared to day 0. ↓ and ↑: decrease and increase.

**Table 3:** Antihyperlipidemic effect of crude methanol seed extract and fraction (CMEDT and MFDT) in streptozotocin-induced diabetic rats.

Group	Treatment (twice daily)	Changes in serum lipid profile in mg/dl				
		T. chol.	TG	HDL	LDL	VLDL
A	CMEDT 50 mg/kg body weight	104.20 ± 31.83^*∗∗*^↓	75.16 ± 4.39^*∗∗*^↓	26.22 ± 0.30	62.95 ± 2.41	15.03 ± 0.88^*∗∗*^↓

B	CMEDT 100 mg/kg body weight	110.80 ± 4.72	85.34 ± 4.83	33.32 ± 1.59^*∗∗*^↑	60.41 ± 5.11	17.07 ± 0.97

C	MFDT 50 mg/kg body weight	111.40 ± 3.71	79.06 ± 5.288^*∗∗*^↓	25.56 ± 2.47	70.03 ± 4.81	15.81 ± 1.06^*∗∗*^↓

D	MFDT 100 mg/kg body weight	106.40 ± 3.67	78.28 ± 3.78^*∗∗*^↓	33.82 ± 1.77^*∗∗*^↑	56.92 ± 2.17^*∗∗*^↓	15.66 ± 0.76^*∗∗*^↓

E	Glibenclamide 10 mg/kg body weight (positive control)	101.20 ± 1.24^*∗∗*^↓	69.40 ± 1.75^*∗∗*^↓	34.04 ± 2.48^*∗∗*^↑	53.28 ± 1.19^*∗∗*^↓	13.88 ± 0.35^*∗∗*^↓

F	Negative (untreated) control	121.20 ± 4.14^*∗*^↑	97.00 ± 3.19^*∗*^↑	25.20 ± 0.40^*∗*^↑	76.60 ± 4.22^*∗*^↑	19.40 ± 0.64^*∗*^↑

G	Normal control	89.20 ± 4.69	76.00 ± 2.45	36.40 ± 1.70	37.60 ± 5.84	15.20 ± 0.49

*F*-ratio		7.374	5.303	7.595	9.705	5.303
*p* value		0.000	0.001	0.000	0.000	0.001

Values are mean ± SEM; *n* = 5; ^*∗*^*p* < 0.05 when compared with normal control; ^*∗∗*^*p* < 0.05 when compared with negative (untreated) control. ↓ and ↑: decrease and increase.

**Table 4:** Atherogenic index and percentage protection of CMEDT and MFDT.

Group	Treatment (twice daily)	Changes in serum lipid profile in mg/dl		Atherogenic index (AI)	% protection
		T. chol.	HDL		
A	CMEDT 50 mg/kg body weight	104.20 ± 31.83	26.22 ± 0.30	2.97	22.01

B	CMEDT 100 mg/kg body weight	110.80 ± 4.72	33.32 ± 1.59	2.33	38.85^*∗*^

C	MFDT 50 mg/kg body weight	111.40 ± 3.71	25.56 ± 2.47	3.36	11.81

D	MFDT 100 mg/kg body weight	106.40 ± 3.67	33.82 ± 1.77	2.15	43.57^*∗*^

E	Glibenclamide 10 mg/kg body weight (positive control)	101.20 ± 1.24	34.04 ± 2.48	2.19	42.52^*∗*^

F	Negative (untreated) control	121.20 ± 4.14	25.20 ± 0.40	3.81	0

G	Normal control	89.20 ± 4.69	36.40 ± 1.70	1.98	48.03

Values are mean ± SEM; *n* = 5; ^*∗*^% protection as compared to untreated control group.

**Table 5:** Effect of crude methanol seed extract and fraction (CMEDT and MFDT) on kidney function parameters in streptozotocin-induced diabetic rats.

Group	Treatment (twice daily)	Changes in serum urea/creatinine	
		Urea (mg/dl)	Creatinine (*µ*mol//l)
A	CMEDT 50 mg/kg body weight	73.06 ± 0.86	44.2 ± 0.00^*∗*^↓
B	CMEDT 100 mg/kg body weight	78.12 ± 0.90	44.2 ± 0.00^*∗*^↓
C	MFDT 50 mg/kg body weight	79.84 ± 4.01	45.97 ± 1.08
D	MFDT 100 mg/kg body weight	100.4 ± 0.93^*∗∗*^↑	44.2 ± 0.00^*∗*^↓
E	Glibenclamide 10 mg/kg body weight (positive control)	96.66 ± 0.55^*∗∗*^↑	44.2 ± 0.00^*∗*^↓
F	Negative (untreated) control	74.22 ± 6.14^*∗*^↑	44.2 ± 0.00^*∗*^↓
G	Normal control	40.48 ± 1.87	47.80 ± 1.10

*F*-ratio		44.732	5.868

*p* value		0.000	0.000

Values are mean ± SEM; *n* = 5; ^*∗*^*p* < 0.05 when compared with normal control; ^*∗∗*^*p* < 0.05 when compared with negative (untreated) control. ↓ and ↑: decrease and increase.

**Table 6:** Phytochemical analysis result of *Dennettia tripetala* seed.

Constituents	Inference
Flavonoids	−
Antraquinone glycosides	−
Anthracene glycosides	−
Alkaloids	+++
Saponins	+++
Tannins	−
Resins	+
Proteins	+
Carbohydrate	+++
Reducing sugars	+++
Hydrolysis test for glycosides	++
Cyanogenetic glycosides	−
Fat & oils	−
Steroids	−
Terpenoids	−
Acidic compounds	Neutral
Cardiac digitoxose	−

*Key*. −: absent; +: present; ++: moderately present; +++: abundantly present.
